# Paediatric Renal Stone Management in Africa: A Scoping Review and Analysis of Current Practices

**DOI:** 10.7759/cureus.56819

**Published:** 2024-03-24

**Authors:** Samuel O Davis, Abdulahi Zubair, Chiagoziem Anyakora, Martin C Igbokwe, Zahraddeen Haladu, Adetola F Ajibade, Olumide Noah, Christian Agyeman, Kenneth Oisamokhai, Obinna Enemoh, Praise Ikuborije, Emeka S Nwokeocha, Oghenofuafo Ajari, Isaac Adejala, Vévé M Mastaki, Oluwatosin Akinro

**Affiliations:** 1 Urology, Surgery Interest Group of Africa, Lagos, NGA; 2 Surgery, Zenith Medical and Kidney Centre, Abuja, NGA

**Keywords:** calculi, paediatric, africa, renal stones, kidney

## Abstract

Renal stones are solid deposits formed from minerals and salts that develop within the kidneys and urinary tract. While the condition is more common among adults, children and even infants can also be affected. There is an increasing incidence of paediatric renal stones in Africa alongside multiple challenges faced in managing the condition. This scoping review aimed to provide an overview of the management modalities of paediatric renal stones in Africa. This study utilised Systematic Reviews and Meta-Analyses extension for Scoping Reviews (PRISMA-ScR) checklist. A systematic search was conducted in three electronic databases: PubMed, African Journal Online (AJOL), and Google Scholar, with 1,180 articles curated. After extensive examination, 10 articles satisfied the inclusion criteria. The review found that calcium oxalate stones were the most prevalent type, accounting for 34.03% of cases, followed by whewellite stones and ammonium urate stones. The most frequent location for stones was the kidney, and the most common symptom was pain. Abdominopelvic ultrasound was the most frequently utilised investigation. Of the 886 patients managed for renal stones, 75.4% were managed surgically, 2.9% medically, and 21.7% spontaneously resolved without intervention. This review identifies opportunities for improving the management of paediatric renal stones in Africa, including the need for standardised diagnostic and treatment protocols and the development of evidence-based guidelines tailored to the African context. Overall, this scoping review provides valuable insights into the patterns and management of paediatric renal stones in Africa and highlights the need for further research to improve the management of this condition in the region.

## Introduction and background

Renal stones are solid deposits formed from minerals and salts that develop within the kidneys and urinary tract. While the condition is more common among adults, children and infants can also be affected [1]. These stones formation is typically due to an oversaturation of the urine with oxalate, calcium, uric acid, or cysteine, which gradually accumulate and form crystals that increase in size over time, leading to characteristic symptoms [2]. Renal stones typically present with symptoms such as flank pain, haematuria, nausea, vomiting, or obstruction, which may require emergency department visits. However, these symptoms are uncommon in young children, who may present with abdominal pain, vomiting, fever, and signs of urinary tract infection (UTI) [3]. Smaller children often present with non-specific signs, while some cases are asymptomatic and detected incidentally [4].

The initial evaluation involves taking a detailed medical history, including diet and fluid intake, family history, medications, and anatomic and metabolic disorders. The National Institute for Health and Care Excellence (NICE) recommends ultrasound as the first-line imaging for diagnosis, with non-contrast CT reserved for cases where there is uncertainty about the diagnosis after an ultrasound scan [5].

Management modalities depend on the child’s functional status, size, composition, and location of the stone [6]. Pain control, adequate hydration, and management of infection are essential in the acute setting. Antibiotics should be considered in signs of infection, while surgical intervention may be necessary when stones are causing an obstruction [2]. This study examines the patterns of paediatric renal stones in Africa and the management modalities among clinicians practising in the continent.

## Review

Methods

Eligibility Criteria

We included all full-text articles that reported data on the management of paediatric renal stones in Africa. Excluded from our review were reviews, meta-analyses, abstracts, conference presentations, commentaries, case reports, and letters to the editors. Additionally, we excluded studies that were not published in English or those not conducted in human populations. In cases where multiple studies involved the same or overlapping patient population, we selected the most recent study. The following studies were excluded for this reason [7-11].

Information Sources, Selection, and Data Charting

The Preferred Reporting Items for Systematic Reviews and Meta-Analyses extension for Scoping Reviews (PRISMA-ScR) checklist was utilised in this study. We conducted a thorough literature search using PubMed, African Journal Online, and Google Scholar to gather relevant articles on the management of paediatric renal stones in Africa from inception to the present day. This search was conducted on January 19, 2023. Our search strategy was developed collaboratively by the authors. The search results were imported into Rayyan.ai, a systematic review software, for deduplication and screening purposes. Articles were selected based on their relevance to the management of paediatric renal stones in Africa. The search strategy utilised in this study is outlined in Table 1.

**Table 1:** Search strategy

Database	Search	Papers
PubMed	(Paediatric Renal Stones) AND (Management) AND (Africa) ("Paediatric Renal Stones"[MeSH Terms] OR ("Paediatric Renal Stones"[All Fields] AND "Management"[All Fields]) OR "Paediatric Renal Stones Management"[All Fields]) AND ("africa"[MeSH Terms] OR "africa"[All Fields])	19
Google Scholar	Management of Paediatric Renal Stones in Africa	980
African Journal Online	Management of Paediatric Renal Stones in Africa	181 results were seen. No additional study was identified.

Critical Appraisal of Data Sources and Evidence

We screened titles and abstracts of all articles generated following the keyword search, with a focus on articles that explicitly addressed paediatric renal stone management in African countries. Full-text screening followed this initial phase. Discrepancies were discussed and resolved with a third reviewer. We then extracted relevant data from the identified studies, including the study ID, title, year of publication, country of origin, sample size, mean age, signs and symptoms at presentation, stone site and type, investigations performed, management approaches (such as spontaneous resolution, surgical management, and medical therapy), mortality rates, and complications.

Synthesis of Results

We collected and recorded all extracted data into a secure Excel® database (Microsoft, Redmond, WA, USA) that was encrypted for privacy. To populate each section in the results, we grouped studies that reported specific domains related to management and collated the extracted data.

Results

Prisma Flowchart

**Figure 1:**
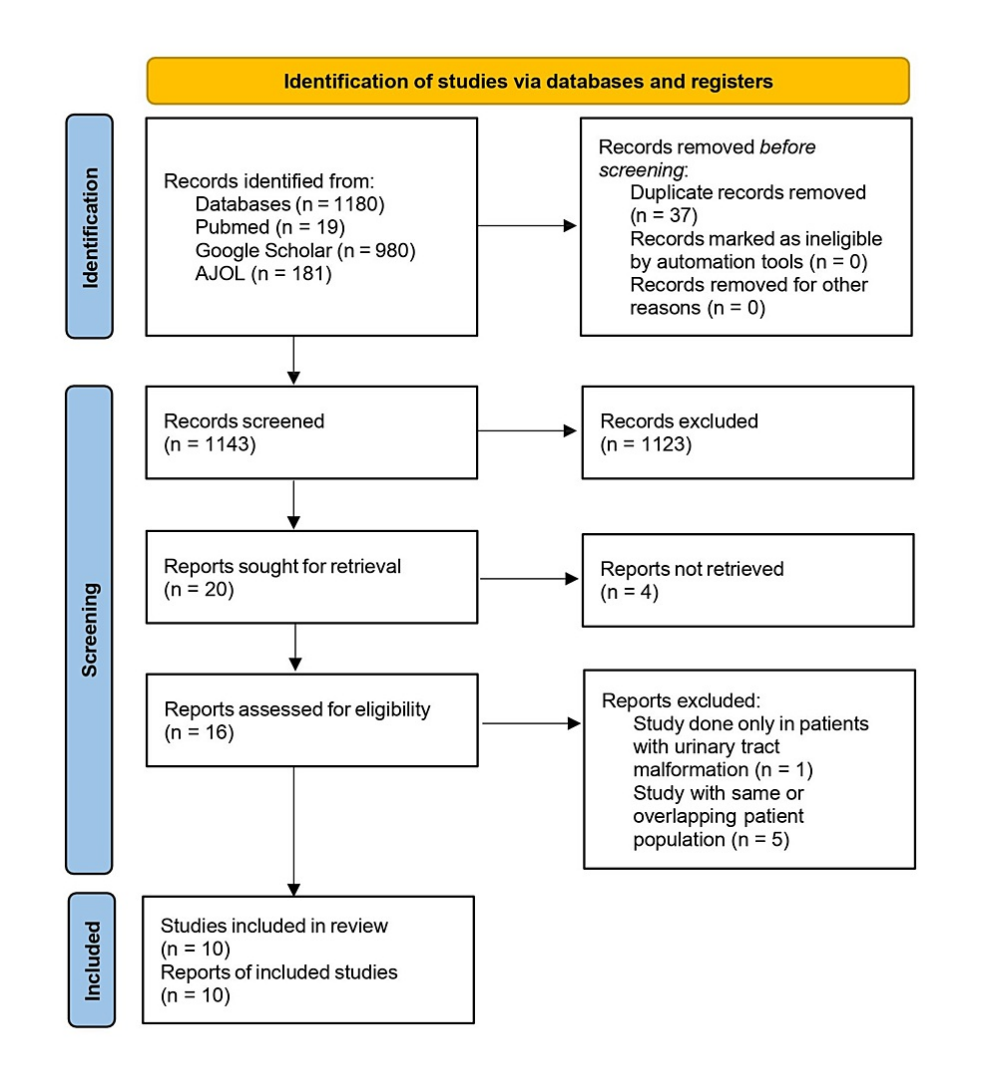
PRISMA flowchart of the study PRISMA: Preferred Reporting Items for Systematic Reviews and Meta-Analyses; AJOL: African Journal Online.

A total of 1,143 studies were screened after the initial database search and deduplication, and only 16 studies met the eligibility criteria. After undergoing full-text screening, five studies [7-11] were excluded as they had the same or overlapping patient population and one study [12] was also excluded as it was carried out only in patients with renal tract malformations. Ten studies were used in this review. The PRISMA selection process is shown in Figure 1.

Study Characteristics

The studies were conducted from 2002 to 2022 in seven different African countries (Nigeria, Ethiopia, Egypt, Morocco, Tunisia, Sudan, and Somalia, with Nigeria contributing the most with three studies). A total of 1,872 patients were included in the study. The study characteristics are represented in Table 2.

**Table 2:** Study characteristics

Country of Origin	Studies (n)	Author	Mean Age (years)	Male (n)	Female (n)	Sample size (n)	Year of Publication
Nigeria	3	Hassan and Mabogunje [13]	4.25	22	0	22	1993
		Chinda et al. [14]	6.7	46	10	56	2011
		Abubakar et al. [15]	6.9	63	4	67	2004
Egypt	2	Badawy et al. [16]	8.63	371	129	500	2012
		Shahin [17]	10.45	13	7	20	2002
Tunisia	1	Alaya et al. [18]	8.6	184	126	310	2012
Ethiopia	1	Metaferia and Shimelis [19]	NA	54	9	63	2014
Sudan	1	Elfadil et al. [20]	4.96	129	46	175	2010
Somalia	1	Eraslan et al. [21]	12.7	127	100	227	2022
Morocco	1	Meiouet et al. [22]	8	302	130	432	2019

Presenting Symptoms

In six of the studies included in this research, presenting signs and symptoms of patients with urinary stones were reported. The most frequently reported symptom was pain, which was present in 65% of cases (290 cases) and described as either abdominal or flank pain. Followed by haematuria reported in 191 cases, nausea and vomiting were the least commonly reported symptoms, with an incidence rate of six cases.

Location of Stone

In the analysed studies, seven out of 10 reported the location of the stones, with the kidney being the most frequent location, accounting for 65% (802) of cases. This is followed by the ureter with 15.9% (195 cases), and 15% (184 cases) of stones located in the bladder. The remaining 4% of cases were stones in the urethra (29 cases) and in multiple locations (10 cases).

Chemical Composition of Stone

Various studies investigated the stone composition, revealing the most prevalent type as calcium oxalate stones (356 cases, 34.03%). Additionally, whewellite stones accounted for 133 cases (12.72%), while 203 cases (19.41%) had ammonium urate stones, and 18 cases (1.72%) had weddellite stones. Carbapatite stones were found in 56 cases (5.35%), struvite stones in 57 cases (5.45%), and anhydrous uric acid stones in 40 cases (3.82%). Cysteine stones were identified in 28 cases (2.68%), with one case (0.01%) of ammonium urate stones. Calcium phosphate stones were present in 30 cases (2.87%), serum urate acid in 13 cases (1.24%), vaterite stones in nine cases (0.86%), and phosphate stones in 12 cases (1.15%). Magnesium ammonium phosphate hexahydrate stones were reported in 86 cases (8.22%).

Investigation Leading to Diagnosis

Various investigations diagnosed urinary stones and ruled out other conditions. In 90% of the studies, subjects had an abdominopelvic ultrasound as the most common test. Intravenous urogram (IVU) and urine cultures were conducted in eight studies, with *Escherichia coli* and *Klebsiella* species being the most commonly isolated organisms in the urine cultures. Urinalysis and plain abdominal X-rays were conducted among subjects in six studies. Computed tomography of the kidneys, ureters, and bladder (CT KUB) and renal function tests were conducted in three and two studies, respectively. Twenty-four-hour urine collection was reported in only one study.

Treatment Modality

Disease conditions linked to paediatric renal stones in Africa are classified as infective, metabolic, and anatomical. Among these, coexisting metabolic disorders are the most prevalent (130 cases, 47.1%), followed by infective causes (88 cases, 31.9%) and coexisting anatomical disorders (58 cases, 21%). Two infectious conditions are responsible for stone formation: diarrhoea, more common at 97.7%, and concomitant schistosome fibrosis contributing only 2.3%.

**Figure 2:**
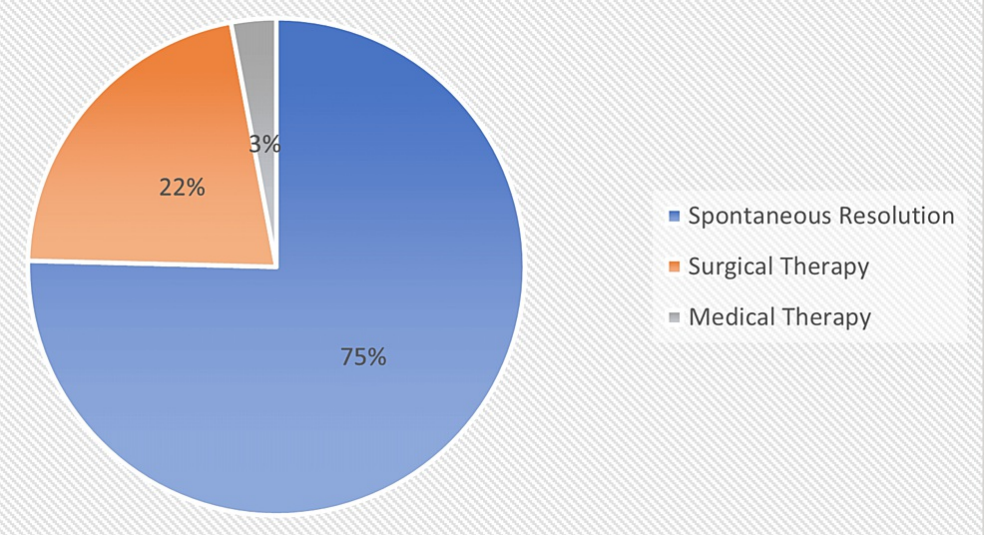
Mode of management of paediatric renal stones Original work of the authors. Of 886 patients managed for renal stones, 668 (75%) were managed surgically, 26 (3%) medically, and 192 (22%) spontaneously resolved. The figures were rounded to whole numbers for clarity: 75.4% and 2.9%.

From the total number of 886 patients who were managed for renal stones, 668 (75%) were managed surgically, 26 (3%) medically, and 192 (22%) spontaneously resolved without intervention. This is represented by the pie chart in Figure 2.

**Figure 3:**
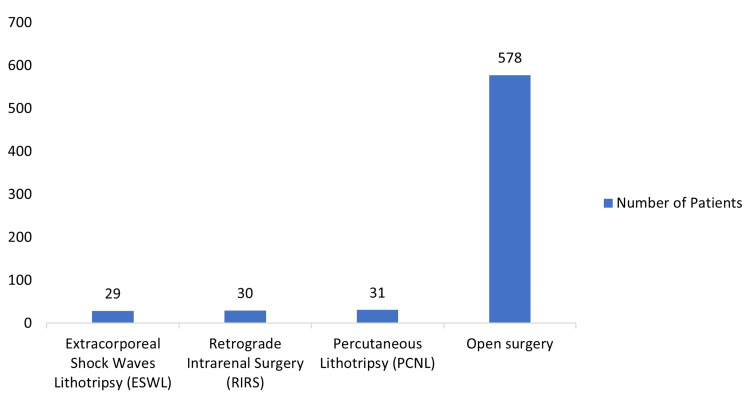
Type of surgical intervention Original work of the authors. Among the patients who had surgical intervention, open surgery accounted for 86.5% (578 patients), percutaneous lithotripsy in 4.6% (31 patients), retrograde intrarenal surgery in 4.5% (30 patients), and the least preferred method being extracorporeal shock wave lithotripsy (ESWL) in 4.3% (29 patients).

Surgical management of paediatric renal stones in these studies was the most commonly used management. The various types of surgeries employed were retrograde intrarenal surgery, percutaneous lithotripsy, open surgery, and extracorporeal shock waves lithotripsy. Among the patients who underwent surgical intervention, open surgery accounted for 86.5% (578 patients), followed by percutaneous lithotripsy and retrograde intrarenal surgery at 4.6% (31 patients) and 4.5% (30 patients), respectively. The least preferred method was extracorporeal shock wave lithotripsy (ESWL), which constituted 4.3% (29 patients) of surgical interventions for managing stones in African children, and all these are represented on the bar chart in Figure 3 below.

Of the 29 patients who underwent ESWL, 18 (90%) achieved stone-free status, while two (10%) were considered failures, with one requiring ureteroscopy and the other managed through open ureterolithotomy. Medical therapy is the least utilised method for managing renal stones in African children, accounting for only 3% (26 patients) of the treatment modalities employed. Among the 26 patients treated with medical therapy, six were managed using D-penicillamine, with only two patients experiencing successful outcomes. Out of the total patients seeking care, the number of patients managed through watchful waiting for the spontaneous passage of renal stones was 192 (21.7%).

Complication

The studies analysed in this research yielded a total of 358 cases of urinary tract infection as a complication of paediatric urolithiasis. *Escherichia coli* and *Klebsiella* spp. were the most common organisms implicated. Additionally, a single study reported 20 cases of haematuria, while another study reported 29 cases of renal scarring complications.

Recurrence Rate and Mortality

Urolithiasis recurred in 186 cases from three studies. There was no mortality recorded.

Discussion

This study reviewed 10 studies from seven countries over the period of 2002 to 2022 with 1,872 patients being examined. This study sought to examine the different prevalent management practices in African urological centres, their outcomes, and complications. This review demonstrated that calcium oxalate is the commonest renal calculi in the subjects studied. This is similar to studies [1,3,23] done in the past and can be attributed to consumption of oxalate-rich foods, malabsorption [24], and metabolic abnormalities such as hypercalciuria and hyperoxaluria [18]. Pain was the predominant symptom in these patients, and upon investigation, most of the stones were located in the kidneys, similar to the study by Rizvi et al. [25].

The commonest investigative tool recorded in the management of paediatric renal stones is abdominopelvic ultrasound scan, in 90% of the cases, despite the commonest stone implicated being calcium oxalate stones. Other investigative tools recorded were abdominal X-rays, urinalysis, CT KUB, and urine cultures. Abdominopelvic ultrasound scan was largely used likely because it is safe, very accessible in the developing world and relatively cheap, and this aligns with the recommendation of the European Association of Urology (EAU) for primary diagnosis of urolithiasis, following a detailed history and physical examination [6]. CT KUB, despite its sensitivity to calcium oxalate stones, was used in only six studies, likely due to concerns about radiation exposure, limited accessibility in rural areas, and cost [26]. The EAU recommends a non-contrast helical CT scan as the gold-standard test (97% sensitivity, 96% specificity) for diagnosing urolithiasis, but it should be used when ultrasound and abdominal radiograph are inconclusive due to radiation concerns in children. Other noted complications included renal scarring and haematuria. In the examined publications, there was no mortality resulting from renal calculi.

In this study, 75.4% of patients underwent surgery, making it the most common treatment modality in African paediatric urological centres. About 21.7% of cases resolved spontaneously (watchful waiting), while 2.9% were managed medically. Although decisions about the management of urolithiasis in children are based on the number, size, location, stone composition, and anatomy of the urinary tract, technological advancements mean that most stones are managed with endoscopic techniques that are less invasive [27]. The most common approaches employed in the surgical management of patients in our study group were retrograde intrarenal surgery, percutaneous lithotripsy, open surgery, and extracorporeal shock waves lithotripsy, with open surgery being the most commonly used approach with 86% of patients managed with open surgery. This is in contrast to studies from the developed parts of the world where minimally invasive approaches such as percutaneous nephrolithotomy (PCNL), ureteroscopy, percutaneous (suprapubic) cystolithotripsy, perurethral cystolithotripsy, and extracorporeal shock-wave lithotripsy (ESWL) are the most used surgical methods used in managing paediatric urolithiasis [28,29]. Medical management with a thiol-containing agent, D-penicillamine, was given to six patients. Copelovitch recommends initial pain control with narcotic analgesics (morphine sulphate) and/or nonsteroidal drugs (ketorolac), rehydration, and the use of agents that may promote the passage of stones and reduce symptoms (medical expulsive therapy), such as alpha-adrenergic blockers (tamsulosin) [30].

Previous research on the subject has been extensive, including scoping reviews, systematic reviews, and meta-analyses that provide evidence. However, this study is unique as it focuses solely on African literature to generate evidence for local practices, aiming to avoid plagiarism. Further studies on a geographic basis may be necessary to establish region-specific guidelines and practices for improved patient care. The study has limitations, including a small sample size, limited inclusion of African studies impacting generalisation, and the possibility of missing relevant non-English articles. Additionally, outcome analysis is limited due to insufficient management disclosure in some papers and the absence of a standardised outcome measurement method.

## Conclusions

Due to the evolving trends in paediatric renal stones globally, this study sought to provide evidence of patterns of paediatric renal stones as well as management modalities amongst clinicians practising in Africa with data from seven different countries. This review suggests that abdominopelvic ultrasound scans are widely used for diagnosis due to their safety profile and low cost, while CT KUB is not as widespread as in the US and Europe. Calcium oxalate is the predominant renal stone in the continent with the kidneys being the most common location. More studies need to be done on this subject to capture many of the regional and geographic peculiarities of urologic practice in Africa.
